# What Role Do Traditional Beliefs Play in Treatment Seeking and Delay for Buruli Ulcer Disease?–Insights from a Mixed Methods Study in Cameroon

**DOI:** 10.1371/journal.pone.0036954

**Published:** 2012-05-18

**Authors:** Koen Peeters Grietens, Elizabeth Toomer, Alphonse Um Boock, Susanna Hausmann-Muela, Hans Peeters, Kirezi Kanobana, Charlotte Gryseels, Joan Muela Ribera

**Affiliations:** 1 Institute of Tropical Medicine, Department of Public Health, Antwerp, Belgium; 2 PASS International, Tessenderlo, Belgium; 3 Aide aux Lépreux Emmaüs-Suisse, Yaoundé, Cameroun; 4 University of Leuven, Centre for Sociological Research, Belgium; 5 Institute of Tropical Medicine, Department of Biomedical Sciences, Antwerp, Belgium; University of Utah, United States of America

## Abstract

**Background:**

Victims of Buruli ulcer disease (BUD) frequently report to specialized units at a late stage of the disease. This delay has been associated with local beliefs and a preference for traditional healing linked to a reportedly mystical origin of the disease. We assessed the role beliefs play in determining BUD sufferers’ choice between traditional and biomedical treatments.

**Methods:**

Anthropological fieldwork was conducted in community and clinical settings in the region of Ayos and Akonolinga in Central Cameroon. The research design consisted of a mixed methods study, triangulating a qualitative strand based on ethnographic research and quantitative data obtained through a survey presented to all patients at the Ayos and Akonolinga hospitals (N = 79) at the time of study and in four endemic communities (N = 73) belonging to the hospitals’ catchment area.

**Results:**

The analysis of BUD sufferers’ health-seeking behaviour showed extremely complex therapeutic itineraries, including various attempts and failures both in the biomedical and traditional fields. Contrary to expectations, nearly half of all hospital patients attributed their illness to mystical causes, while traditional healers admitted patients they perceived to be infected by natural causes. Moreover, both patients in hospitals and in communities often combined elements of both types of treatments. Ultimately, perceptions regarding the effectiveness of the treatment, the option for local treatment as a cost prevention strategy and the characteristics of the doctor-patient relationship were more determinant for treatment choice than beliefs.

**Discussion:**

The ascription of delay and treatment choice to beliefs constitutes an over-simplification of BUD health-seeking behaviour and places the responsibility directly on the shoulders of BUD sufferers while potentially neglecting other structural elements. While more efficacious treatment in the biomedical sector is likely to reduce perceived mystical involvement in the disease, additional decentralization could constitute a key element to reduce delay and increase adherence to biomedical treatment.

## Introduction

In a recent paper, Hotez et al. [1] stress the importance of addressing the devastating effects that neglected tropical diseases have on their victims. As one such neglected tropical disease, Buruli ulcer disease, bears with it a high social and economic burden for its sufferers [2], [3]. Its causative agent, *Mycobacterium ulcerans,* is an environmental mycobacterium endemic to restricted foci throughout the tropics and directly related to stagnant or slow-flowing water [4]. BUD is a poorly understood disease that has emerged dramatically since the 1980s. The disease is mostly found in rural areas located near wetlands and slow-moving rivers, especially in those prone to flooding and that are often associated with rapid environmental change [5].

Unlike leprosy and tuberculosis, caused by organisms belonging to the same family as BUD, which are characterized by person-to-person transmission, inoculation of Mycobacterium ulcerans into the subcutaneous tissues likely occurs through environmental contact, although the mode of transmission is still not entirely clear [5]. The agent produces a potent toxin known as mycolactone, which destroys cells in the subcutis leading to the development of large skin ulcers [4]. Though most cases of the disease occur in children ages 4–15, all ages and sexes are affected [5]. A review of risk factors worldwide concluded that most studies identify poor wound care, failure to ware protective clothing, and living and working near bodies of water as risk factors [6].

Major advances have been made in the management of the disease with the introduction of rational antibiotic therapy; nevertheless, surgery remains important for BUD treatment. In early stages of infection, surgery is curative and highly cost effective since it requires only a simple excision followed by an immediate closure. At later stages, wide excisions are needed to stop the infection and to prevent relapse at the same site [7]. Successful clinical outcome therefore requires that patients initiate treatment promptly and adhere to treatment regimens [8].

Similar to other neglected tropical diseases, BUD sets into motion a spiral of impoverishment that is difficult to escape. Furthermore, since BUD often results in irreversible physical disabilities, the disease takes a heavy toll on affected patients and their households [3]. Prompt and appropriate treatment, however, can minimize if not completely bypass most of the lasting physical and social side effects of the disease, such as physical malformations, loss of schooling, amputation and severe reductions in productivity. Nonetheless, victims of BUD often report to specialized units at a late stage of the disease [8], presenting an apparent contradiction that is poorly understood.

Research and interventions directed at neglected tropical diseases still largely neglect the social, ecological, and other contextual factors that allow diseases to persist in specific populations. These factors, however, are key to uncovering the reasons behind delayed arrival at biomedical health facilities and behind therapy choice. The last few years have witnessed an increase in awareness of socio-cultural factors influencing delay and access to hospital treatment with various studies focusing on elements guiding treatment choice for BUD sufferers [9], [10]. A reportedly shared finding from previous research summarized in the WHO factsheet [8], is that “in developing countries, socio-cultural beliefs and practices strongly influence the health-seeking behaviours of people affected by BU. The first recourse is often traditional treatment”. As a consequence, “most patients seek treatment too late”. The factsheet further refers to the high direct and indirect costs, possible fear of stigma due to disfiguration from surgery and concerns about scars and possible amputations as reasons for a reported preference for traditional healing. Considering this finding, we analysed BUD-patients’ therapeutic itineraries in the region of Ayos and Akonolinga in Central Cameroon to verify whether local socio-cultural beliefs and practices determined patients’ therapeutic itineraries. If so, it would be expected that these beliefs determine patients’ preference for biomedical or traditional treatment; that both treatment options are perceived as mutually exclusive and incompatible; and finally, that the perceived mystical aetiology of the disease and consequent traditional treatment would constitute a primary and direct source of delay. This article presents the findings of this analysis and the role beliefs play in determining treatment choice and delay. Data from this study evaluating the economic and social impact of the cost burden of BUD were published elsewhere [3].

## Methods

### Study Site and Population

The present study was conducted in the region of Ayos and Akonolinga in Central Cameroon where BUD was first documented in 1969 in 47 cases. The endemic region was later identified as an area stretching along the Nyong River and some of its tributaries for approximately 100 km in length and 10–30 km in width with a population of 98,500 [4]. A study by Noeske *et al.* [4] conducted in 2001 identified an overall prevalence rate of 0.44% constituting active and inactive Buruli ulcer cases in the surveyed area. The highest prevalence of active cases found in a particular settlement was 8%. Disease prevalence was higher in villages closer to the Nyong river. A survey carried out by Um Boock in 2004 [11] further detected new foci of BUD outside of the previously established endemic region, particularly in other areas of the Central Province and in the provinces of the East and Southwest. At the national level, 930 cases were detected.

Biomedical treatment for BUD in the study region is provided at the Ayos and Akonolinga Hospitals, which have specialized BUD programmes following WHO treatment guidelines. The study region is populated by various segmented identity subgroups of Beti, primarily, but not limited to, the Yebekolo, the Ewondo, the Sso and the Maka. These groups rely mainly on subsistence farming and fishing for sustenance. Pouillot *et al.* identified swamp wading and wearing short lower body clothing while farming as main risk factors in the study area [12]. Protective factors included bed net use, washing clothes, and using leaves as traditional treatment or rubbing alcohol when hurt.

### Research Strategy

The research design consisted of a mixed methods study based on methodological triangulation, combining qualitative data from focused ethnography and quantitative data gathered using a standardized questionnaire. Field work was conducted in both community and clinical settings for a period of four months, between November 2005 and February 2006, three of which were spent at the Ayos and Akonolinga hospitals and one in the selected endemic communities of Eyess, Edou, Ebanda and Ngoulemakong, all belonging to the catchment areas of the respective hospitals. Considering the focalized character of BUD infection rates, [13] restricted local and geographical units were selected for analysis.

### Qualitative Data

#### Data collection

Qualitative data were gathered during ethnographic fieldwork. The emphasis on qualitative data collection for the first strand of the study was required given the exploratory nature of the first phase of research and the sensitive content of research questions related to traditional healing, the acceptability of hospital treatment and the possible mystical origin of the disease.

The following data collection techniques were used:

##### Participant observation

Participant observation consisted of participating in everyday activities at the hospital and community settings, observing events in their usual context and carrying out reiterated informal conversations and interviews. During fieldwork at the hospital settings, special emphasis was placed on the analysis of factors related to patient satisfaction with biomedical treatment (including the doctor-patient relationship, practical and financial implications of hospitalization, the role of caregivers, etc.); on the perceived aetiology of BUD in relation to treatment choice; and, on gaining an in-depth understanding of factors directly guiding treatment itineraries. Participant observation was an essential component of the field research as this facilitated in building up confidence with informants and in acquiring an in-depth understanding of more sensitive subjects such as sorcery involvement in the illness and healing processes. The observation of patients’ daily activities provided an opportunity for reiterated informal conversations with key respondents, leading to some pivotal insights regarding the doctor-patient relationship, the importance of caregivers and the complexities of the disease’s causality.

##### Interviews

Interviews were held in all selected locations and, when possible, recorded and fully transcribed. When the interviewer(s) considered that recording or note taking in the presence of the respondent was inappropriate due to the sensitive nature of the subjects discussed, the required informality of the interview, the respondents’ preferences or other limitations, the conversation was not recorded. The content of the interview was then written down immediately after the interview. Interviews were mostly carried out in French, the official language in the region and the local *lingua franca*. When villagers were not proficient in the language, interviews were carried out in local languages (*Maka, Yebekolo*, etc.) with the help of a translator/mediator. Interviews were almost exclusively carried out in private settings to increase confidentiality.

##### Group discussions

Group discussions were held with health staff, hospital patients at the hospital and family clusters in villages. Group discussions were not recorded since initial discussions revealed that the formality of recording decreased the reliability of the response for delicate subjects such as the doctor-patient relationship and topics related to local beliefs.

#### Sampling

Sampling was purposive. Following the principle of gradual selection, informants were theoretically selected (in accordance with emerging results/theory) and categorized in relation to relevant criteria (such as gender, age, religion, ethnicity, locality, hospital or traditional treatment for BUD, economic activities, etc.) to allow for maximum variation. In addition, critical cases were continuously selected and analysed. Snowball sampling (using participants to identify additional cases) was used in order to increase respondents’ confidence in the research team and consequently reduce response bias (i.e. new respondents are more likely to be confident in the researchers’ trustworthiness after an acquaintance’s referral).

#### Data analysis

In accordance with the research strategy, data gathering and analysis were concurrent and data analysis was a continuous, flexible and iterative process. Preliminary data, collected through various techniques, were intermittently analysed after which further research was conducted to confirm or refute temporary results until saturation was reached and the data could furthermore be theoretically supported. Raw data were processed in their textual form and coded to generate and/or identify analytical categories or themes for further analysis. The systemization and analysis of all qualitative data was carried out with N/Vivo Qualitative Analysis software (QSR International Pty Ltd. Cardigan UK).

### Quantitative Data

#### Data collection

After the initial qualitative research strand of the study, data were more systematically gathered and standardized through a quantifiable half-open structured questionnaire in both hospital and community settings. The questionnaire was designed to quantify relevant variables from the qualitative strand and test related hypotheses. The questionnaire centred on treatment itineraries and therapy choice as well as on the cost burden of BUD (see [3]).

#### Sampling

At the Ayos and Akonolinga Hospitals, 79 clinically confirmed hospitalized BUD patients were included in the sample, representing all patients in treatment during the four-month period of the study from November 2005 to February 2006. In the communities, 73 patients were included in the sample, representing –according to local health specialists, BUD sufferers’ families and other key informants in the affected communities– all active and inactive Buruli ulcer cases living in the selected communities at the time of study.

#### Data analysis

Quantitative data were entered in Excel and analysed in SAS (SAS Institute Inc., SAS Campus Drive, Cary, North Carolina 27513, USA).

### Ethical Considerations

The study was approved by the ethical committee of the Ministry of Health, Cameroon (No. 0123/ARRO/MSP/DPSPL). Local health authorities and community leaders were informed about the study objectives and procedures for data collection. Regarding the ethnographic data collection, all interviewers followed the Code of Ethics of the American Anthropological Association (AAA) [14]. All interviewees were informed before the start of the interview about project goals, the topic and type of questions, their right to refuse being interviewed, to interrupt the conversation at any time, and to withdraw any given information during or after the interview, and the intended use of the results for scientific publications and reports to health authorities. Oral consent was preferred -and approved by the Ministry of Health’s ethical committee- since the interviewees were not put at any risk of being harmed physically or psychologically by participating in the study and because the act of signing one’s name when providing information during informal conversations could be a potential reason for mistrust [15]. All interviews were carried out by the principal investigator and a co-researcher/witness of the consent procedure and, when required, with the help of a translator. When consent was not obtained, participants were automatically excluded from the sample.

## Results

### Perceived Aetiology

BUD, mostly known in the Ayos and Akonolinga region as *atom,* and more broadly as an incurable wound (*plaie inguérissable*), has various perceived origins, which categorize the disease as either a mystical illness (*maladie mystique*) or a natural illness (*maladie simple*).

#### Mystical infection

Mystical infection with BUD is caused by an infraction against *mvoe* (a traditional concept expressing both social order and health) or by sorcery. First, as an infraction against social order, the disease is frequently linked to theft and trespassing on agricultural plots. Fields are frequently protected by magical charms or *bian* (also known as ‘*fetish*’), in which various diseases, including BUD, can be contained. For those unfortunate enough to trespass on, steal from or simply urinate or spit on such protected plots, the *fetish* will infect the transgressor with the illness(es) held within it. However, in cases of baby/infant victims of BUD where transgressions against social order are improbable if not impossible, a second mystical aetiology exists based on human causal involvement. In this scenario, the illness is believed to be cast (*lancé*) at others by means of sorcery.

Sorcery operates through the intervention of an agent or force of the invisible world identified as *evou* (or ‘*evu*’), which can be understood as an anti-social, often malignant force, which is an active agent in sorcerers (or *nnem*) and can be passively available in others. Sorcerers and certain healers (*ngengan*) have the capacity to “see” what is invisible to other people: “we see only during the day, the sorcerer sees at night and therefore has ‘four eyes’”. Protection and healing from sorcery attacks also depend on the strength of each individual’s *evou*. To gain mastery over the sorcery-related illness, both sorcerer and healer engage in ‘night battles’, the outcome of which determines the patient’s health. Sorcerers are often accused of the so-called ‘eating of human flesh’, referring to the belief that they mystically sacrifice the limbs and even lives of others, often family members themselves, in return for greater power and prosperity. Not coincidentally, the slow progression with which BUD spreads is likened to the sorcerer gradually consuming the limbs or lives of his victims.

#### Natural infection

Aside from mystical infection, the disease can also have a so-called ‘natural’ origin –i.e. without human agency and mystical involvement. The most frequently cited natural causes for BUD are insect bites, specifically by the horsefly known as ‘*ossun*’, and sustaining generally minor wounds and/or developing infections of varying degrees. The biomedical explanation for BUD as water-related and caused by a ‘*microbe*’ is also generally known and, when perceived to be a natural infection, accepted.

#### Double causality

Despite the above-mentioned and clearly distinct perceived origins of the disease, natural and mystical aetiologies are often interchangeable or linked. This process is known as double causality and refers to an illness having both natural and mystical derivations. As such, even though BUD can be perceived to be naturally transmitted by a microbe, it can simultaneously be believed that the insects transmitting this microbe can be ‘sent’ by a sorcerer to harm the victim -revealing a second and mystical level of causality. Double causality is also apparent when the ‘natural’ categorization of the disease fails to adequately explain the aetiology and/or progression of the disease. For instance, when biomedical explanations fail to respond to questions such as why some people are infected by BUD and others are not despite living in the same community or even household, people seek other answers. Consequently, understanding the biomedical explanations for BUD, as outlined in health education messages and by public health officials does not necessarily rule out human involvement or the possibility that natural infection is a consequence of infractions of social rules or sorcery.

Moreover, beliefs are dynamic. Despite sufferers’ previously held beliefs and available information, the perceived aetiology can alter in accordance with the effectiveness of biomedical or traditional treatments. The prolonged nature of the illness and treatment, the difficulty of the healing process and recidivism can lead to assumptions about possible mystical involvement even for sufferers who were convinced of the natural origin of their illness at the onset of symptoms.

The mechanism of double causality also explains how health staff at local hospitals can share patients’ *evou* beliefs and yet still stand behind biomedical treatment as well as how medical staff recommend traditional healing on occasion when biomedical treatment fails repeatedly.

### Treatment Options

Various treatment options are available for BUD-sufferers:

#### Home treatment

Home treatment mostly consists of one, or a combination, of the following options: (i) applying an inexpensive salve (e.g. *le Chat Blanc*) to alleviate symptoms attributed to insect bites or common abscesses; (ii) consuming antibiotics (and/or painkillers) purchased at pharmacies or local health centres; (iii) using traditional herbs and leaves as remedies.

#### Traditional healing

BUD can be traditionally treated either by a local traditional healer or by an ‘ex-patient healer’; the latter being a non-specialist healer who has either been a victim him/herself of the disease or has observed the healing process closely. The traditional healing process can include one or several of the following steps, largely depending on the aetiology of the illness and on the specialization of the healer him/herself: (i) *Divination.* Divination is used to see into the invisible (magical) world and determine the cause of illness (i.e. infractions of *mvoe*, natural causes, curses, etc.) and the appropriate course of treatment. (ii) *Confession.* When applicable, the sufferer is asked to confess the wrongdoings that may have evoked the disease to his ancestors. He must then voice his regret for his transgressions and express his willingness to cooperate with the healer in order to facilitate the healing. (iii) *Lavage du corps.* The *lavage du corps*, or cleansing of the body, represents the purifying phase of healing whereby the sufferer’s body is washed with water and/or the blood of a sacrificed animal (usually a rooster). (iv) *Interdictions.* Interdictions during treatment vary from healer to healer but most commonly call for the prohibition of eating fresh meat and fish and having sexual relations (these prohibitions apply to the sufferer and the healer as well as to visitors during the treatment period). On exceptional occasions, the proscriptions include refraining from greeting people (in order not to attract more bad luck in the sufferer’s vulnerable state); avoiding the consumption of salt and oil; and, abstaining from other practices such as touching others’ belongings. It is believed that flouting the imposed interdictions stops and even reverses the healing process. These aspects often lead to the *de facto* isolation of the sufferers due to the treatment requirements, as illustrated by the quote: “*Atom* really is the illness of isolation” (Village Chief Edou). (v) *Treatment of the ulcer*. The ulcer itself is usually treated with a combination of herbs, and often with the use of tree bark or other natural substances in various thermo-regulation therapies (applying hot bark to the ulcer or immersing the ulcer and encircling areas in near boiling/scalding water). (vi) *Reintegration*. To conclude the healing process, the healer prepares the food forbidden to the sufferer during treatment to signify the end of the illness and to symbolize his reintegration into society.

Many of the above mentioned treatment phases are linked to magico-religious beliefs and therefore are more so the domain of the traditional healers. ‘Ex-patient healers’ tend to focus primarily on treating the ulcer itself rather than on the magico-religious elements associated with the disease.

#### Biomedical treatment

Biomedical treatment is available at the Ayos and Akonolinga Hospitals, sponsored by *Fairmed (*formerly *Aide aux Lépreux Emmaüs Suisse)* and *Médicins Sans Frontières*, respectively. At the time of study, both programs offered free-of-charge in-patient treatment, following WHO-guidelines, and supplementary aid consisting of free meals served once or twice a day (depending on the institution), complementary accommodations for in-patients and their caretakers for the duration of their stays, supplementary schooling (at Ayos Hospital) and the free but irregular provision of basic materials for everyday needs, such as soap, bandages and sheets.

### Health-seeking Itineraries

#### Number of health encounters

Patients at the Ayos and Akonolinga Hospitals presented a mean of 3 health encounters, with consequent treatment for BUD, prior to arriving at the specialized hospitals (range of 0 to 13 encounters). In the selected communities, respondents presented a mean of 2 health encounters prior to their current treatment (of any kind) (range 0 to 12 encounters) (Table 1).

**Table 1:** Health-seeking itineraries of patients in hospitals and endemic communities.

	Hospital		Endemic Villages	
	%	n	%	n
Nr of health encounters prior to arriving at current treatment(incl. specialised BU unit)				
One health encounter	25,3	19	32,8	18
Two health encounters	32	24	43,3	24
Three health encounters	14,7	11	9,0	5
> Three health encounters	28,0	21	14,9	26
* Total*	*100*	*79*	*100*	*73*
* Mean (range)*	*3 (0–13)*		*2 (0–12)*	
Alternation of treatment in hospital patients with more than one health encounter	%	n	%	n
Alternated between sectors	80	60	71,6	48
Did not alternate	20	15	28,4	19
* Total*	*100*	*75*	*100*	*67*

#### Treatment kind and perceived aetiology

At the time of study, notably almost half (48,1%) of all hospital patients attributed their BUD to mystical causes. 26,6% stated that their BUD was naturally transmitted and an additional 17,4% simply did not know the cause of their illness (7,6% was missing) (Table 2).

**Table 2:** Aetiology of BUD and first treatment options.

	Hospital setting (N = 79)		Endemic villages (N = 73)	
	%	n	%	n
Aetiology of BUD				
Natural	26,6	21	26,0	19
Mystical	48,1	38	56,2	41
Don’t know	17,4	14	17,8	13
Missing	7,6	6	0,0	0
* Total*	*100*	*79*	*100*	*73*
First treatment option				
Home treatment	27,8	22	24,7	18
Biomedical treatment	43,0	34	20,5	15
Traditional healing	27,8	22	53,4	39
Missing	1,3	1	1,4	1
* Total*	*100*	*79*	*100*	*79*
First treatment option excludinghome treatment				
Biomedical treatment	56,0	31	72,2	39
Traditional healing	44,0	25	27,8	15
* Total*	*100*	*56*	*100*	*54*

#### Alternation and combination of treatments

The analysis of the therapeutic itineraries of BUD sufferers with more than one health encounter (93,4% of all sufferers) revealed that 80% of hospital patients and 71,6% of BUD sufferers in local communities had alternated between biomedical and traditional treatment.

#### Compatibility of biomedical and traditional treatment

Qualitative data (especially based on observing traditional treatment) revealed the compatibility and inter-changeability of both kinds of treatment. The use of biomedicine during traditional healing, especially in the intake of antibiotics and pain-relief medicine in combination with herbal treatment (involving heat therapy with bark and leaves) was common and indicated a perception of compatibility between both treatment kinds. Another notable example of this crossover was illustrated when traditional healers in two of the four endemic communities at the time of study were treating victims of BUD that originated from perceived natural causes. Similarly, hospital patients did not limit their treatment options to the biomedical field but, after healing, often opted for ‘*blindage*’. *Blindage* consists of a ritual carried out by a traditional healer, assuring mystical protecting for ex-patients against future BUD infections once they are back in their communities.

### Additional Factors Influencing Health-seeking Behaviour

Although the beliefs discussed above can influence health-seeking itineraries, more compelling factors were identified that determined patients’ treatment paths, indicating that the choice of treatment was not decided upon solely with consideration to disease aetiology. Evident from the qualitative analysis of patients’ itineraries, the following factors were paramount in deciding treatment:

#### Effectiveness of treatment

The length and complexity of patients’ itineraries pointed to a determined search for effective treatment. Some patients faced complete social isolation, the loss of social relations and economic and professional ruin in their search for effective treatment. In the words of one patient: “I have searched for healing even among the pygmies just because it was one more option” (Adult patient at Ayos Hospital).

#### Place of treatment

Treatment within or outside of the BUD victim’s community was one of the decisive factors in determining treatment choice. Treatment outside of the community, whether biomedical or traditional, usually placed an overwhelming financial and social burden on the victim and on his/her household as it either implied constant (and, hence, generally expensive) traveling to receive treatment or social isolation for the patient who was required to stay without relatives at the place of treatment. For many respondents, specialized BUD hospital treatment was not located in or near their communities which signified a series of additional determining costs: (i) Transportation costs: when treatment was not local, transportation costs frequently represented an additional burden for the patient, but even more so for caretakers who would continuously have to travel back and forth between BUD patient’s place of treatment and their residence in order to continue to meet work obligations, social responsibilities (work groups, savings groups) and household tasks (i.e. attending to other children). (ii) Feeding costs: the cost of providing food for a patient could be minimized through the provision of agricultural products from the household’s slash and burn fields. However, this coping strategy was only feasible when regular visits to the patient were also feasible, such as when treatment was carried out in the general vicinity of the patient’s community. Moreover, in many cases, food provision to the traditional healer was a form of payment for the treatment, minimizing the financial burden of the disease for the patient and the household. (iii) Productivity loss: though the productivity loss (lost earnings during the course of treatment [3]) of BUD patients was arguably comparable in the hospital and community settings, that of caregivers was greatly affected by the location of the patient’s treatment. In the hospital setting, caregivers, especially for the very young and in certain cases for the elderly, were required to aid in the patients’ daily care (washing, cleaning, cooking) since those services were not provided by the hospital and could not always be carried out by the patients themselves. Nevertheless, when the patient received treatment in the vicinity of his community, the caregiver could combine his/her daily economic activities (such as working on fields) with the patient’s care without a serious impact on productivity. However, as alluded to earlier, this combination of caring for the patient and sustaining work obligations frequently became overwhelming if the patient’s treatment was not local, as was often the case when a patient was hospitalized. Accordingly, the place of treatment was a decisive element guiding both BUD patients’ treatment choice (often in favour of traditional healing) as well as affecting their adherence to a given treatment.

#### Difficulties of symptom recognition

When looking at the reported time it took patients to act upon the presented symptoms and seek treatment, we see that 74.0% of all community respondents reported to start treatment (including home treatment, traditional healing or biomedical treatment) within three weeks of the onset of symptoms (Table 3) while 11% delayed treatment for more than 3 months. The principal reason for delaying the decision to seek specialized care was aptly phrased by one informant: “*if we have to go to the hospital with every little bump we have…*”. During the first stage of infection, symptoms are frequently mistaken for insect bites and especially for everyday skin infections or abscesses.

**Table 3:** Delay prior to treatment seeking among hospital patients.

	%	n
Time before seeking treatment		
Reporting immediate action	19,2	14
<3 weeks	54,8	40
>3 weeks (<3 months)	13,7	11
>3 months	11,0	8
Missing	1,4	1
* Total*	*100*	*79*

#### Acceptability of treatment

A last decisive set of factors that influenced health-seeking behaviour were those categorized under treatment acceptability. While certain patients mentioned the fear of skin grafting or other inconveniences associated with biomedical treatment, the main determinant factor cited was directly related to the perceived inhospitable hierarchical doctor-patient relationship at hospital settings. This dynamic is most clearly illustrated in the paraphrased words of one ex-BUD hospital patient: “I spent months there at the hospital, but I could only endure so much. They treat you like a dog. But I’m a *man*. So finally I had no choice but to leave” (BUD sufferer, Ngoulemakong). While traditional healing was generally not subject to this perception, in certain cases, patients claimed that the lack of respect by medical staff at the hospital led to treatment abandonment.

## Discussion

The analysis of BUD sufferers’ health-seeking behaviour showed extremely long and complex therapeutic itineraries, including various attempts and failures both in the biomedical and traditional fields. Contrary to expectations, nearly half of all hospital patients attributed their illness to mystical causes while traditional healers admitted patients they perceived to be infected by natural causes. Moreover, both patients in hospitals and in local communities often combined elements of both types of treatments while looking for effective healing (i.e. antibiotics during traditional healing and mystical protection against recidivism for hospital patients). These findings show that both treatment processes were not mutually exclusive but often linked and, as such, they call into question both the current dichotomous framework (biomedical versus traditional/mystical aetiology) used to explain health-seeking itineraries and the very importance of aetiological beliefs for treatment seeking.

Patients’ therapeutic itineraries cannot be understood without insight into the mechanism of double causality (i.e. an illness having both natural and mystical derivations), the interchangeability and frequent compatibility of the two treatment kinds, the dynamic nature of aetiological beliefs, and insight into decisive factors that impact treatment choice. Although its implications on disease control are rarely taken into account, various other studies have stressed the importance of double causality in relation to other diseases. Hausmann and Muela show how people in rural Tanzania are well aware that malaria is caused by parasites. However, they also claim that these parasites can be mystically ‘hidden’ during biomedical diagnosis, leading to possibly fatal delays in finding the appropriate treatment. Likewise, sorcery can produce ‘fake parasites’, once more leading to erroneous diagnosis delayed treatment [16]. Similarly, in the South-African context, Thomas explains that, while biomedical narratives on HIV provide information on the virus and how it develops, they do not provide people with an explanation of *why* they became infected in the first place [17]. Thomas claims that this double causal layer is essential in understanding people’s perception of the illness and, consequently, their perception and response to HIV-treatment. Even the presence of intestinal worms can be associated with multiple causalities as a study among the Luo in Kenya shows. In this context, intestinal worms can be either biomedical “intruders”, requiring expulsion by “hospital medicine”, or “positive agents” of digestion that, nevertheless, require herbal remedies when they cause discomfort and illness due to eating taboo foods or to witchcraft [18]. Our research, likewise, shows that the perceived origins of respondents’ BUD often had both natural and mystical causal layers simultaneously. For instance, though respondents often believed that they were infected with Buruli ulcer by an insect bite, many of them also stated that this insect was intentionally sent to them through sorcery. As such, efforts at disseminating biomedical explanations for BUD, as outlined in health education messages, can impact treatment seeking and delay, but this is not necessarily the case since additional mystical elements can be present in natural aetiologies.

Despite these insights into the complexity of belief systems with relation to disease, our results show that the beliefs about the origins of Buruli ulcer did not determine treatment choice. The fact that sufferers infected naturally with Buruli ulcer often turned to traditional treatment, and vice versa, contradicts the assumption that aetiology dictates treatment kind. Moreover, the frequent alternation and combination of both types of treatment for BUD further disputes the view that perceived aetiology directly leads to a preference for one treatment kind over the other. Finally, sufferers’ illness interpretations would often change when they either came into to contact with bio-medical explanations for the disease or when the prolonged nature of the disease and the difficulty of attaining healing and/or relapse/recidivism of the illness led them to assume mystical involvement. Accordingly, the fact that beliefs concerning the disease’s aetiology were dynamic once more strongly suggests that a direct causal relationship between beliefs and treatment choice cannot be confirmed in this setting.

**Figure 1:**
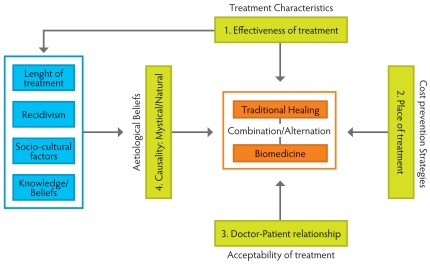
Determinant factors for treatment choice in BUD patients’ health itineraries (numbered in order of importance).

It is apparent in these findings that determinants other than belief systems are more decisive in guiding people’s treatment choice (Figure 1). The place of treatment, related costs and psychosocial well-being, proved to be decisive for treatment choice and delay. BUD sufferers attempted to minimize or completely circumvent the debilitating costs associated with Buruli ulcer treatment. The fact that local treatment was largely able to accomplish this goal was cited by respondents as a major reason why traditional treatment was often preferred to biomedical treatment. When asking what happens to people who cannot afford to treat an illness that requires hospital treatment, the answer was clear: “then you die in the forest”. This shows people’s use of ‘cost prevention strategies’ (strategies employed in order to prevent the accumulation of debilitating costs -contrary to ‘cost management strategies’, dealing with how costs are managed; i.e. borrowing, labour substitution, selling assets [19]) and indicates that these strategies largely guided patients’ and their families’ health-seeking decisions. In this sense, delay in seeking treatment in the biomedical sector is a strategy to prevent escalating costs as well as a consequence of the difficulty in initially distinguishing BUD symptoms from everyday insect bites or abscesses. Moreover, the overall difficulty of finding successful treatment alongside the expected inhospitable reception at the hospital was an important factor contributing to late stage arrival at specialized facilities and treatment abandonment [3].

Other studies have highlighted factors affecting BUD victims’ delay in seeking appropriate treatment and treatment choice, including distance to the hospital [3], [12], [13], [20]; high medical costs [2]; hospitalization time [3], [12], [13], [20]; stigma [12], [13], [21]; fear of mutilation, amputation and skin grafting [12], [13]; and, social isolation [3]. Several of the above-mentioned factors (i.e. distance, high medical costs, hospitalization time) can be more broadly conceptualized as cost prevention strategies, rather than as isolated factors, further corroborating our findings. Nonetheless, a prominent perception still holds that preference for traditional healing due to erroneous beliefs is a major factor fostering delay. It has, indeed, been shown in many countries that Buruli ulcer transmission is widely associated with sorcery and witchcraft (Ghana [10], Benin [21], Cameroon [11], Congo [22]) along with a marked tendency for BUD victims to seek treatment at the traditional healer’s. Nevertheless, our study concludes that attributing delay and treatment choice to ‘erroneous’ beliefs or a mere lack of knowledge constitutes a stark over-simplification of BUD health-seeking behaviour and is counterproductive in acquiring a scientifically sound framework to interpret treatment choice and delay.

In terms of the research strategy applied, certain decisive insights into traditional healing and the complexities of disease causality and treatment choice could only be obtained through reiterated conversations of informal nature with key respondents and the building up of trust with patients and community members during participant observation. We argue that the same findings could not have been obtained with more formal research methods and without an emphasis on fieldwork and qualitative research methods for the first strand of the study. Conversely, a limitation of the study design was that not all relevant factors related to treatment itineraries and choice could be further quantified.

### Conclusion

As the long and complex health-seeking itineraries of BUD sufferers illustrate, victims of debilitating tropical diseases are not ignorant of the consequences of not finding appropriate treatment for their illnesses. They actively seek to understand their illness, the healing process and the underlying causes of their misfortune [19]. The persistent ascription of delay to beliefs has significant implications for policy planning since it places the burden of responsibility for delay and treatment choice directly on the shoulders of BUD sufferers, as opposed to on the health system itself, therefore neglecting other possible structural elements that may influence delay. In this sense, while more efficacious treatment in the biomedical sector would likely reduce perceived mystical involvement in the disease, additional decentralization and the reduction of the duration of hospital stays, responding to people’s use of cost prevention strategies, could be key in increasing adherence to biomedical treatment.

## References

[1] Hotez PJ, Molyneux DH, Fenwick A, Ottesen E, Erlich Sachs S (2006). Incorporating a Rapid-Impact Package for Neglected Tropical Diseases with Programs for HIV/AIDS, Tuberculosis, and Malaria.. PLoS Med 3(5):.

[2] Asiedu K, Etuaful S (1998). Socio-economic Implications of Buruli Ulcer in Ghana: A Three-Year Review.. *Am J Trop Med Hyg,*.

[3] Peeters Grietens K, Um Boock A, Peeters H, Hausmann-Muela S, Toomer E (2008). “It Is Me Who Endures but My Family That Suffers”: Social Isolation as a Consequence of the Household Cost Burden of Buruli Ulcer Free of Charge Hospital Treatment.. PLoS Negl Trop Dis 2(10):.

[4] Noeske J, Kuaban C, Rondini S, Sorlin P, Ciaffi L (2004). Buruli Ulcer Disease in Cameroon Rediscovered.. *Am J Trop Med Hyg,*.

[5] Merrit RW, Walker ED, Small PLC, Wallace JR, Johnson PDR (2010). Ecology and Transmission of Buruli Ulcer Disease: A Systematic Review.. PLoS Negl Trop Dis.

[6] Jacobsen K, Padgett J (2010). Risk Factors for Mycobacterium ulcerans infection.. *Int J Infect*.

[7] Porten K, Sailor K, Comte E, Njikap A, Sobry A (2009). Prevalence of Buruli Ulcer in Akonolinga Health District, Cameroon: Results of a Cross Sectional Survey.. *PLoS Negl Trop Dis*.

[8] WHO (2007). http://www.who.int/mediacentre/factsheets/fs199/en/Accessed.

[9] Stienstra Y, van der Graaf W T, Asamoa K, van der Werf TS (2002). Beliefs and Attitudes toward Buruli ulcer in Ghana.. *Am J Trop Med Hyg*.

[10] Mulder A, Boerma R, Barogui Y, Zinsou C, Johnson RC (2008). Healthcare seeking behaviour for Buruli ulcer in Benin: a model to capture therapy choice of patients and healthy community members.. *Trans R Soc Trop Med Hyg*.

[11] Um Boock A (2004). Lepra Aid Internal Report..

[12] Pouillot R, Matias G, Wondje CM, Portaels F, Valin N (2007). Risk Factors for Buruli Ulcer: A Case Control Study in Cameroon.. *PLoS Negl Trop Dis*.

[13] Johnson P, Stinear T, Small P, Plushke G, Merritt R (2005). Buruli Ulcer (M. ulcerans Infection): New Insights, New Hope for Disease Control.. *PLoS Med*.

[14] American Anthropological Association (2008). http://www.aaanet.org/committees/ethics/ethcode.htm.

[15] American Anthropological Association (2004). http://www.aaanet.org/stmts/irb.htm.

[16] Hausmann-Muela S, Muela Ribera J, Tanner M (1998). Fake malaria and hidden parasites – The ambiguity of malaria.. *Anthropology and Medicine,*.

[17] Thomas F (2008). Indigenous Narratives of HIV-AIDS: Morality and Blame in a Time of Change.. *Medical Anthropology*, *27*(3),.

[18] Geissler PW (1998). ‘Worms are our life’, part I: Understandings of worms and the body among the Luo of western Kenya.. *Anthropology & Medicine, 5*(1),.

[19] Russell S (2004). The Economic Burden of Illness for Households in Developing Countries: A Review of Studies Focusing on Malaria, Tuberculosis, and Immunodeficiency virus/acquired immunodeficiency syndrome.. *Am J Trop Med Hyg,*.

[20] Ajoulat I, Johnson C, Zinsou C, Guédénon A, Portaels F (2003). Psychosocial aspects of health seeking behaviours of patients with Buruli ulcer in southern Benin.. *Trop Med Int Health,*.

[21] Muela Ribera J, Peeters Grietens K, Toomer E, Hausmann-Muela S (2009). A Word of Caution against the Stigma Trend in Neglected Tropical Disease Research and Control.. *PLoS Negl Trop Dis*.

[22] Kapay Kibadi K, Boelaert M, Kayinua M, Minuku J, Muyembe-Tamfum J (2009). Therapeutic itineraries of patients with ulcerated forms of Mycobacterium ulcerans (Buruli ulcer) disease in a rural health zone in the Democratic Republic of Congo.. http://dx.doi.org/10.1111/j.1365–3156.2009.02324.x.

